# Evaluation of NxTAG Respiratory Pathogen Panel and Comparison with xTAG Respiratory Viral Panel Fast v2 and Film Array Respiratory Panel for Detecting Respiratory Pathogens in Nasopharyngeal Aspirates and Swine/Avian-Origin Influenza A Subtypes in Culture Isolates

**DOI:** 10.1155/2017/1324276

**Published:** 2017-08-29

**Authors:** K. H. Chan, K. K. W. To, P. T. W. Li, T. L. Wong, R. Zhang, K. K. H. Chik, G. Chan, C. C. Y. Yip, H. L. Chen, I. F. N. Hung, J. F. W. Chan, K. Y. Yuen

**Affiliations:** ^1^Department of Microbiology, The University of Hong Kong, Pokfulam, Hong Kong; ^2^State Key Laboratory for Emerging Infectious Diseases, The University of Hong Kong, Pokfulam, Hong Kong; ^3^Carol Yu Centre for Infection, The University of Hong Kong, Pokfulam, Hong Kong; ^4^Department of Microbiology, Queen Mary Hospital, Pokfulam, Hong Kong; ^5^Department of Medicine, The University of Hong Kong, Pokfulam, Hong Kong; ^6^The Collaborative Innovation Center for Diagnosis and Treatment of Infectious Diseases, The University of Hong Kong, Pokfulam, Hong Kong

## Abstract

This study evaluated a new multiplex kit, Luminex NxTAG Respiratory Pathogen Panel, for respiratory pathogens and compared it with xTAG RVP Fast v2 and FilmArray Respiratory Panel using nasopharyngeal aspirate specimens and culture isolates of different swine/avian-origin influenza A subtypes (H2N2, H5N1, H7N9, H5N6, and H9N2). NxTAG RPP gave sensitivity of 95.2%, specificity of 99.6%, PPV of 93.5%, and NPV of 99.7%. NxTAG RPP, xTAG RVP, and FilmArray RP had highly concordant performance among each other for the detection of respiratory pathogens. The mean analytic sensitivity (TCID50/ml) of NxTAG RPP, xTAG RVP, and FilmArray RP for detection of swine/avian-origin influenza A subtype isolates was 0.7, 41.8, and 0.8, respectively. All three multiplex assays correctly typed and genotyped the influenza viruses, except for NxTAG RRP that could not distinguish H3N2 from H3N2v. Further investigation should be performed if H3N2v is suspected to be the cause of disease. Sensitive and specific laboratory diagnosis of all influenza A viruses subtypes is especially essential in certain epidemic regions, such as Southeast Asia. The results of this study should help clinical laboratory professionals to be aware of the different performances of commercially available molecular multiplex RT-PCR assays that are commonly adopted in many clinical diagnostic laboratories.

## 1. Introduction

Respiratory tract infections (RTIs) are caused by various pathogens that are often indistinguishable from one another by clinical diagnosis. RTIs are a major cause of mortality, morbidity, and hospitalization, especially in children aged below five years, and pose significant economic burden [1–6]. Most RTIs are caused by viruses. Common respiratory viruses include influenza virus, adenovirus, parainfluenza virus, respiratory syncytial virus (RSV), human coronaviruses, human metapneumovirus (hMPV), and rhinovirus [7, 8].

Rapid and accurate identification of the causative pathogens of RTIs can help guide treatment decisions, possibly reducing the length of hospital stays and associated healthcare costs. Moreover, it aids in epidemiologic tracking of local outbreaks or epidemics by implementing proper infection control measures and administering effective antimicrobial therapy. Rapid laboratory identification of RTI pathogens has been associated with up to 50% reduction in hospital stays, 30% reduction in the inappropriate use of antibiotics, and 20% reduction in unnecessary diagnostic tests and procedures [9]. Multiplexed molecular assays that can concurrently detect multiple pathogens from the same sample in a single reaction have been increasingly adopted in clinical microbiology laboratories in the recent years [10]. Several studies have shown that these molecular tests have superior diagnostic capacity and cost-effectiveness of molecular tests when compared to conventional and other diagnostic methods [11, 12].

For influenza viruses, rapid molecular tests based on polymerase chain reaction (PCR) using primers that target the relatively conserved matrix (M) gene are frequently used for achieving rapid and accurate laboratory diagnosis. However, variations in the gene and amino acid sequences in different influenza virus subtypes due to rapid mutations of the viral RNA polymerase may affect the results of these tests. With the emergence of human infections due to the swine-origin influenza A H3N2 variant (H3N2v) virus in North America in 2010 and more recently the avian-origin influenza A H7N9 and H5N6 in China [13–15], it would be important to ascertain the analytical sensitivities of these rapid tests for the novel influenza viruses [16, 17]. In a previous study, we have demonstrated that xTAG RVP has lower sensitivity for detection of H7N9 in patient samples and culture isolates [16, 17].

NxTAG Respiratory Pathogen Panel (RPP), CE-IVD (Luminex Molecular Diagnostics, Toronto, Canada), is a recent new qualitative multiplex molecular test for the detection of nucleic acids from multiple respiratory viruses and bacteria extracted from respiratory specimens. It detects 22 viral and bacterial targets including influenza A virus, influenza A virus subtype seasonal H1, influenza A virus subtype seasonal H3, influenza A virus subtype swine-origin H1N1pdm09, influenza B virus, RSV A and B, parainfluenza viruses types 1 to 4, adenovirus, hMPV, rhinovirus/enterovirus, human coronavirus- (HCoV-) 229E, HCoV-OC43, HCoV-NL63, HCoV-HKU1, human bocavirus,* Chlamydophila pneumoniae*,* Mycoplasma pneumoniae*, and* Legionella pneumophila* (Table 1). Although there were several studies about the performance of NxTAG RPP using RUO (research use only) assay, the performance of the NxTAG RPP, CV-IVD, for detection of respiratory viruses, particularly the sensitivity and specificity of influenza A subtypes, remains unclear [18–20]. The purpose of this study is to evaluate the NxTAG RPP, CE-IVD, and compare it with Luminex xTAG Respiratory Viral Panel (RVP) Fast v2 (Luminex Molecular Diagnostics, Toronto, Canada) and FilmArray Respiratory Panel (RP) (bioMérieux, Marcy l'Étoile, France), using nasopharyngeal aspirate (NPA) samples obtained from patients with RTIs and culture isolates of different swine and avian-origin influenza A subtypes (H2N2, H3N2v, H5N1, H5N6, H7N9, and H9N2).

## 2. Materials and Methods

### 2.1. Clinical Specimens

The 133 NPA specimens selectively collected from patients with symptoms and signs of RTIs who were managed in Queen Mary Hospital, Hong Kong, and with clinical PCR results were used in this study. These NPA samples were collected into a viral transport medium as described previously [21] and were tested by direct immunofluorescence (DFA) (D^3^® Ultra 8™ DFA Respiratory Virus Screening and Identification Kit, Diagnostic Hybrids, Inc. (Quidel), USA) for influenza A virus, influenza B virus, parainfluenza viruses types 1 to 4, respiratory syncytial virus, human metapneumovirus, and adenovirus, and an aliquot of each NPA was sent to Hong Kong Government Virus Laboratory, the Department of Health, for further testing by RT-PCR for detection of influenza A virus, influenza B virus, parainfluenza viruses types 1 to 4, respiratory syncytial virus, adenovirus, and rhinovirus/enterovirus, as part of the routine clinical diagnostic protocol. Single RT-PCR for detection of human metapneumovirus, human coronavirus- (HCoV-) 229E, HCoV-OC43, HCoV-NL63, HCoV-HKU1, human bocavirus,* Chlamydophila pneumoniae*,* Mycoplasma pneumoniae*, and* Legionella pneumophila* was used as described previously [22–27]. All these PCR results were used as positive reference. The remaining samples were then recruited for NxTAG RVP evaluation. A sufficient quantity of the samples was used for further testing with xTAG RVP and FilmArray RP.

### 2.2. Influenza Isolates

Two swine-origin and four avian-origin influenza A human isolates, namely, H1N1pdm09 (A/415742/09/H1N1), H3N2v (A/Wisconsin/12/2010), H2N2 (A/Asia/57/3), H5N1 (A/Vietnam/3028/04), H7N9 (A/Anhui/1/2013), and H9N2 (A/HK/1073/99), were used in this study. Additionally, an H5N6 avian isolate [H5N6 (A/Great Egret/H5N6/HK/2016)] was also included. The viruses were cultured for 2 days in Madin-Darby Canine Kidney (MDCK) cells with tosyl sulfonyl phenylalanyl chloromethyl ketone- (TPCK-) treated trypsin (2 *µ*g/ml) (Sigma, St. Louis, MO) as previously described [28]. Aliquots of culture supernatant were frozen at −80°C until use. Serial tenfold dilutions of the virus stock with serum-free minimal essential medium (MEM) (Gibco BRL, Grand Island, NY) were tested in NxTAG RPP, xTAG RVP, and FilmArray RP. The viral M gene genome copy numbers per ml of each virus dilution were determined by real-time, quantitative RT-PCR (q-PCR) as previously described with modification [29, 30]. Briefly, 5 *μ*L purified RNA was amplified in a 25 *μ*L reaction containing 0.5 *μ*L Superscript III Reverse Transcriptase/Platinum Taq DNA polymerase (Invitrogen, Carlsbad, California, USA), 0.05 *μ*L ROX reference dye (25 mM), 12.5 *μ*L of 2x reaction buffer, 800 nmol/L forward primer (5′-GACCRATCCTGTCACCTCTGAC-3′), 800 nmol/L reverse primer (5′-AGGGCATTYTGGACAAAKCGTCTA-3′), and probe 200 nmol/L (FAM-5′-TGCAGTCCTCGCTCACTGGGCACG-3′-BHQ1). The thermal cycling conditions were 30 minutes at 50°C for reverse transcription and then 2 min at 95°C for RT inactivation/initial denaturation, followed by 50 cycles of 15 s at 95°C and 30 s at 55°C. All reactions were performed using StepOnePlus Real-Time PCR System (Applied Biosystems, Foster City). For quantitative assay, a reference standard plasmid was prepared by cloning the target M gene amplicon into pCRII-TOPO vector (TOPO™ TA Cloning™ Kit, Invitrogen, San Diego, CA) according to the manufacturer's instructions. The copy number of the standard plasmid was determined based on molar concentration of the plasmid (1 molar is equivalent to about 6.0221415 × 10^23^ copies numbers). A series of 6 log10 dilutions, equivalent to 1 × 10^1^ to 1 × 10^6^ copies per reaction, were then prepared from the reference standard plasmid to generate calibration curves and run in parallel with the test samples. The limit of detection of the M gene qRT-PCR is 10 copies per reaction at 95% confidence level. The same virus dilutions were inoculated onto MDCK cells to determine the 50% tissue culture infective dose (TCID50) using the Reed-Muench method as previously described [28].

### 2.3. Nucleic Acid Extraction

Nucleic acid was detected using easyMAG extraction platform (bioMérieux, Marcy l'Étoile, France) and extracted for NxTAG RPP and xTAG RVP from the specimens as previously described [31]. Total nucleic acid extraction was isolated by using the NucliSENS easyMAG instrument (bioMérieux). Briefly, 10 *μ*l of MS2 internal control was first added to 200 *µ*l of sample. The mixture was added to 2 ml of lysis buffer and was incubated for 10 minutes at room temperature. The lysed sample was then transferred to the well of a plastic vessel with 100 *µ*l of silica. This was followed by automatic magnetic separation. Nucleic acid was recovered in 110 *µ*l elution buffer. By using a parallel specimen (300 *µ*l), nucleic acid extraction, PCR, and detection were performed in FilmArray according to the manufacturer's instructions. The NxTAG RPP incorporates multiplex Reverse Transcriptase-Polymerase Chain Reaction (RT-PCR) with the Luminex proprietary universal tag sorting system on the Luminex platform for detecting respiratory pathogen targets according to the manufacturer's instructions. Briefly, the extracted total nucleic acid is added to preplated Lyophilized Bead Reagents (LBRs) and mixed to resuspend the reaction reagents. The reaction is amplified via RT-PCR and the reaction product undergoes bead hybridization within the sealed reaction well. The hybridized, tagged beads are then sorted and read on the MAGPIX instrument, and the signals are analyzed using the NxTAG Respiratory Pathogen Panel Assay File for SYNCT™ Software (Luminex, Austin, Texas, USA).

### 2.4. Statistical Analysis

Diagnostic sensitivity, specificity, PPV, and NPV of the assays against the positive references and agreement between the assays were calculated by VassarStats: Website for Statistical Computation (http://vassarstats.net).

## 3. Results

### 3.1. Clinical Samples and Performance of NxTAG RPP

A total of 133 NPA specimens were tested in this study. The specimens were collected from 75 males and 58 females with a mean age of 32.5 years (range: 2 months to 99 years of age). Of 133 specimens, 75 positives were detected by DFA and 151 positives were detected by PCR [influenza A virus = 16 (H3 = 12, H1pdm09 = 3, H7 = 1), influenza B virus = 10, parainfluenza virus 1 = 10, parainfluenza virus 2 = 10, parainfluenza virus 3 = 10, parainfluenza virus 4 = 11, RSV = 11, hMPV = 11, adenovirus = 4, rhinovirus/enterovirus = 28, human coronavirus- (HCoV-) 229E = 3, HCoV-OC43 = 6, HCoV-NL63 = 1, HCoV-HKU1 = 5, human bocavirus = 9,* Chlamydophila pneumoniae* = 1,* Mycoplasma pneumoniae* = 4, and* Legionella pneumophila* = 1]. All 133 specimens were evaluated by NxTAG RPP. Overall, 153 pathogens were identified by NxTAG RPP. Ten positives (H1pdm09 × 1, RSV A × 1, P1F1 × 1, adenovirus × 1, EV/rhinovirus × 4, and bocavirus × 2) were considered as false positives when compared with positive reference. The sensitivity, specificity, positive predictive value, and negative predictive value of NxTAG RPP for detection of respiratory viruses were 95.2% [95% confidence level (Cl): 90.4–97.7], 99.6% (95% Cl: 99.3–99.8), 93.5% (95% Cl: 88.4–96.5), and 99.7% (95% Cl: 99.4–99.9), respectively (Table 2).

### 3.2. Performance and Concordance of xTAG RVP and FilmArray RP

Of 133 tested samples, 71 had enough quantity to be used for further testing with xTAG RVP and FilmArray RP. The results of these 71 samples tested by the three assays were then compared. Out of these 71 specimens, 66 were positive and 5 were negative by NxTAG RPP. Both the xTAG RVP and FilmArray RP detected respiratory pathogens in 63 (88.7%) specimens with 96 and 83 pathogens, respectively. The sensitivity, specificity, positive predictive value, and negative predictive value of xTAG RVP and FilmArray RP were 96.9%, 99.9%, 99.0%, and 99.7% and 85.3%, 99.9%, 98.8%, and 98.9%, respectively. When NxTAG RPP was compared with xTAG RVP and FilmArray RP, the Kappa value was 0.97 with 95% CI of 0.95–0.99 and 0.93 with 95% CI of 0.89–0.97, respectively. When xTAG RVP was compared with FilmArray RP, the Kappa value was 0.95 with 95% CI of 0.91–0.98. These results suggested that the three assays were highly concordant as a Kappa value higher than 0.80 indicated concordance.

We have previously shown that xTAG RVP has low sensitivity for detecting H7N9 [16, 17]. In this study, one of the patient specimens with confirmed H7N9 influenza A infection was also not detected by xTAG RVP influenza A but was detected by NxTAG RPP and FilmArray RP influenza A. xTAG RVP has a 1000 and 10 times higher limit of detection for H7N9 than NxTAG RPP and FilmArray RP, respectively (Table 3). These findings show that NxTAG RRP has greatly improved the sensitivity for detecting H7N9 over the xTAG RVP. Besides, NxTAG RPP has also improved sensitivity for detecting other respiratory viruses over xTAG RVP (1 adenovirus and 2 bocavirus). These findings corroborate another recent report on the partial comparison between the NxTAG Respiratory Pathogen Panel Assay and the Luminex xTAG Respiratory Panel Fast Assay v2 [32]. NxTAG RPP detected 12 more pathogens than FilmArray RP in our study, with 50% (6/12) being enterovirus/rhinovirus. The other additional positives detected by NxTAG RPP were adenovirus (2), parainfluenza virus 4b (1), hMPV (1), H1N1pdm (1), and* M. pneumoniae* (1). Overall, 8 positives, hMPV (1), P2 (1), P4 (3), enterovirus/rhinovirus (2), and* M. pneumoniae* (1), were not detected by NxTAG RPP. All these positives indeed had high Ct value (≥35) that may be lower than the limit of detection by NxTAG RPP. Further study should be required if the primers that are suspected do not detect epidemic strains.

Furthermore, the results of 5 samples (4 H1N1pdm 2009 and 1 H7N9) were reported to be “equivocal” by FilmArray RP (Flu A-pan-1 positive, Flu A-pan-2 negative). These 5 specimens were, in fact, true positive as confirmed by other testing methods. These findings indicated that M gene (Flu A-pan-1) was more sensitive than NS1 gene (Flu A-pan-2) for detection of H1N1pdm 2009.

### 3.3. Analytical Sensitivity of Influenza A Culture Isolates

When influenza A virus subtype isolates were tested, the mean (range) analytic sensitivities (viral load log10 copies/ml and TCID_50_/ml) of NxTAG RPP, xTAG RVP, and FilmArray RP were 3.0 (1.8–4.2) and 0.7 (0.0056–3.2); 4.3 (3.0–6.4) and 41.8 (0.56–178); and 3.4 (2.7–4.4) and 0.8 (0.1–1.78), respectively (Table 3). All avian- and swine-origin influenza A strains were detected by M gene in NxTAG RPP, xTAG RVP, and FilmArray RP. The swine-origin H1N1pdm09 was correctly genotyped by NxTAG RPP and FilmArray RP. However, H3N2v and seasonal H3N2 could not be differentiated by NxTAG RPP.

## 4. Discussion

In the recent years, the availability of molecular diagnostic tests has revolutionized the diagnosis and surveillance of infectious diseases and treatment of RTIs. Multiplexed molecular assays simultaneously detect multiple pathogens from the same sample in a single reaction vessel, providing a more comprehensive picture of infection. In this study, the clinical performance of the Luminex NxTAG RPP (CV-IVD) in NPA specimens was found to have high sensitivity (95.2%) and specificity (99.6%) for detection of respiratory viruses. These findings are similar to those reported in recent evaluation studies using NxTAG RPP (RUO) kit by other groups [18–20]. The performances of the NxTAG RPP, xTAG RVP, and FilmArray RP were also compared. Their results were highly concordant, with the highest concordance reported between NxTAG RRP and xTAG RVP [Kappa value = 0.97 (95% Cl: 0.95–0.99)].

According to the manufacturer, M gene (Flu A-pan-1) and NS1 gene (Flu A-pan-2) are used for influenza A typing by FilmArray RP. The result is only interpreted as positive when both genes are positive. The result will be interpreted as “equivocal” when only Flu A-pan-1 is positive, and the system will suggest the users to repeat testing by the same assay or other assays. In this study, the results showed that an “equivocal” result by FilmArray RP is most likely to be a true positive. The correct interpretation of these results should be considered to avoid delay in diagnosis and treatment particularly in patients with severe influenza A virus infection.

In general, the analytical sensitivities of the NxTAG RPP and FilmArray RP for the swine- and avian-origin influenza viruses were comparable, but xTAG RVP had more than one log lower analytical sensitivity than NxTAG RPP and FilmArray RP (Table 3). NxTAG RPP also had higher analytical sensitivity for detecting H5N6 than the xTAG RVP but the same sensitivity as FilmArray RP and higher analytical sensitivity for detecting H3N2v than xTAG RVP and FilmArray RP. All three multiplex assays accurately typed and genotyped the influenza viruses, except for NxTAG RRP that does not distinguish subtyped H3N2 from H3N2v. This finding was further confirmed by testing another H3N2v strain, A/Indiana/08/2011, with 500TCID_50_. A recent study also showed that NxTAG RPP (RUO) only correctly genotyped 95.5% of influenza A virus H1N1pdm09 strains [19].

Rapid multiplex RT-PCR assays for respiratory pathogens detection are important for establishing diagnosis to facilitate the implementation of appropriate treatment and infection control measures. Sensitive and specific laboratory diagnosis of all influenza A virus subtypes is especially essential in certain epidemic regions, such as Southeast Asia. The correct interpretation of these results is also important and would avoid delay in patient management and infection control, especially in patients with severe seasonal influenza A or avian/swine-origin influenza A virus subtype infection. Clinical microbiologists, infectious disease specialists, and laboratory managers should be aware of and understand the different clinical performances of these commercially available molecular multiplex RT-PCR assays that are commonly adopted in many clinical microbiology laboratories. The sensitivity and specificity of these molecular multiplex assays must be predetermined and be able to confidently detect most of the important avian or swine influenza A subtype infection including H5N1 and H7N9 and this capability is particularly important for use in Southeast Asia.

A limitation of this study is that some target numbers are small. This is due to low prevalent disease and may require a longer period in order to collect a higher number of positives. High cost for these multiplex assays is another limitation for us to study more samples.

## 5. Conclusion

In this study, the results showed that NxTAG RPP, xTAG RVP, and FilmArray RP assays had high sensitivity and specificity for diagnosis of respiratory diseases. They are suitable for use in clinical diagnostic laboratories for detection of respiratory pathogens in patients with RTIs. However, awareness should be raised that H3N2 could not be distinguished from H3N2v by NxTAG RPP. Further investigation should be performed if H3N2v is suspected to be the cause of disease.

## Figures and Tables

**Table 1:** Detectable pathogens and targets.

Viruses	NxTAG RPP	xTAG RVP Fast v2	FilmArray RP
Influenza A virus	√	√	√
Subtype (H1, H1pdm09, H3)	√	(H1, H3)	√
Influenza B virus	√	√	√
Respiratory syncytial virus	(A, B)	(A, B)	√
Human coronavirus (229E, OC43, NL63, HKU1)	√	√	√
Parainfluenza viruses 1–4	√	√	√
Human metapneumovirus	√	√	√
Adenovirus	√	√	√
Human bocavirus	√	√	
Human rhinovirus/Enterovirus	√	√	√
*Chlamydophila pneumoniae*	√		√
*Legionella pneumophila*	√		
*Mycoplasma pneumoniae*	√		√
*Bordetella pertussis*			√

Total targets	22	18	20

**Table 2:** Performance of NxTAG RRP for detecting respiratory viruses in nasopharyngeal aspirates.

Viruses	NxTAG Pos/ Ref Pos	NxTAG Neg/ Ref Pos	NxTAG Pos/ Ref Neg	NxTAG Neg/ Ref Neg	Sensitivity % (95% Cl)	Specificity % (95% Cl)	PPV % (95% Cl)	NPV % (95% Cl)
Influenza A	16	0	1	116	100 (75.9–100)	99.1 (94.6–100)	94.1 (69.2–99.7)	100 (96.0–100)
H3	12	0	0	121	100 (69.9–100)	100 (96.2–100)	100 (69.9–100)	100 (96.2–100)
H1pdm09	3	0	1	129	100 (31.0–100)	99.2 (95.1–100)	75 (21.9–98.7)	100 (96.4–100)
H1	0	0	0	133	NA	NA	NA	NA
Influenza B	10	0	0	123	100 (65.5–100)	100 (96.2–100)	100 (65.5–100)	100 (95.5–100)
RSV A	9	0	1	123	100 (62.9–100)	99.2 (94.9–100)	90 (54.1–99.5)	100 (96.2–100)
RSV B	2	0	0	131	100 (19.8–100)	100 (96.4–100)	100 (19.8–100)	100 (96.4–100)
PIF1	10	0	1	122	100 (65.5–100)	99.2 (94.9–100)	99.9 (57.1–99.5)	100 (96.2–100)
PIF2	9	1	0	123	90.0 (54.1–99.4)	100 (96.2–100)	100 (62.9–100)	99.2 (94.9–100)
PIF3	10	0	0	123	100 (65.5–100)	100 (96.2–100)	100 (65.5–100)	100 (96.2–100)
PIF4	8	3	0	122	72.7 (39.0–92.6)	100 (96.2–100)	100 (59.8–100)	97.6 (92.6–99)
Adenovirus	4	0	1	128	100 (39.6–100)	99.2 (95.1–100)	80.0 (29.9–98.9)	100 (96.4–100)
hMPV	10	1	0	122	90.9 (57.1–99.5)	100 (96.2–100)	100 (66.5–100)	99.2 (94.9–100)
HCoV-229E	3	0	0	130	100 (31.0–100)	100 (96.4–100)	100 (31.0–100)	100 (96.4–100)
HCoV-OC43	6	0	0	127	100 (51.7–100)	100 (96.3–100)	100 (51.7–100)	100 (96.3–100)
HCoV-NL63	1	0	0	132	100 (5.5–100)	100 (96.5–100)	100 (5.5–100)	100 (96.5–100)
HCoV-HKU1	5	0	0	128	100 (46.3–100)	100 (96.4–100)	100 (46.3–100)	100 (96.4–100)
EV/rhinovirus	26	2	4	101	92.9 (75.0–98.8)	96.2 (90.0–98.8)	86.7 (68.4–95.6)	98.1 (92.4–100)
Bocavirus	9	0	2	122	100 (62.9–100)	98.4 (93.7–99.7)	81.8 (47.8–96.8)	100 (96.2–100)
*C. pneumoniae*	1	0	0	132	100 (5.5–100)	100 (96.5–100)	100 (5.5–100)	100 (96.5–100)
*M. pneumoniae*	3	1	0	129	75.0 (21.9–98.7)	100 (96.3–100)	100 (31.0–100)	99.2 (95.2–100)
*L. pneumophila*	1	0	0	132	100 (5.5–100)	100 (96.5–100)	100 (5.5–100)	100 (96.5–100)
*B. pertussis*	0	0	0	133	NA	NA	NA	NA

Total	158	8	11^#^	2882	95.2 (90.4–97.7)	99.6 (99.3–99.8)	93.5 (88.4–96.5)	99.7 (99.4–99.9)

EV, enterovirus; HCoV; human coronavirus; hMPV, human metapneumovirus; PIF, parainfluenza virus; PPV, positive predictive value; NPV, negative predictive value; RSV, respiratory syncytial virus, NA; not applicable; Ref, reference; Pos, positive; Neg, negative. ^#^Overall total number of false positives is 10 because influenza A and subtyping were tested for a sample simultaneously.

**Table 3:** Analytical sensitivity of NxTAG RPP, xTAG RVP, and FilmArray RP for detecting influenza A, avian- or swine-origin subtypes.

Influenza A		Limit of detection [viral load (log10 copies/ml); log10 TCID50/ml] of influenza A subtype					
		NxTAG RPP		xTAG RVP		FilmArray RP	
Subtype	Strains	Viral load	TCID_50_	Viral load	TCID_50_	Viral load	TCID_50_
H1N1pdm09	A/HK/415742/09/H1N1	3.0	0.1	3.0	0.1	3.0	0.1
H2N2	A/Asia/57/3	3.7	3.2	3.7	3.2	2.7	0.32
H3N2v	A/Wisconsin/12/2010	2.0	0.1	4.0	10	3.0	1
H5N1	A/Vietnam/3028/04	4.2	0.56	4.2	0.56	4.2	0.56
H5N6	A/H5N6/HK/2016	3.0	1	5.0	100	3.0	1
H7N9	A/Anhui/1/2013	3.4	0.178	6.4	178	4.4	1.78
H9N2	A/HK/1073/99	1.8	0.0056	3.8	0.56	3.8	0.56

Mean		3.0	0.7	4.3	41.8	3.4	0.8
